# The role of color in the perception of three-dimensional shape

**DOI:** 10.1016/j.cub.2022.01.026

**Published:** 2022-03-28

**Authors:** Phillip J. Marlow, Karl R. Gegenfurtner, Barton L. Anderson

**Affiliations:** 1 The University of Sydney, School of Psychology, Sydney, NSW 2006, Australia; 2 Giessen University, Department of Psychology, 23 Ludwigstrasse, 35390 Giessen, Germany

**Keywords:** color, saturation, 3D shape, material, subsurface scattering, shading, translucency, perception

## Abstract

The human visual system can derive information about three-dimensional (3D) shape from the structure of light reflected by surfaces. Most research on single static images has focused on the 3D shape information contained in variations of brightness caused by interactions between the illumination and local surface orientation (“shading”).^1^, ^2^, ^3^, ^4^, ^5^, ^6^ Although color can enhance the recovery of surface shading when color and brightness vary independently,^7^, ^8^, ^9^ there is no evidence that color alone provides any information about 3D shape. Here, we show that the wavelength-dependent reflectance of chromatic materials provides information about the 3D shape of translucent materials. We show that different wavelengths of light undergo varying degrees of subsurface light transport, which generates multiple forms of spatial structure: wavelengths that are weakly reflected generate shading-like image structure, linked to 3D surface orientation, whereas wavelengths that penetrate more deeply into the material are primarily constrained by the direction of surface curvature (convexities and concavities).^10^ Psychophysical experiments demonstrate that the enhanced perception of 3D shape in chromatic translucent surfaces arises from the shading structure generated by weakly reflected wavelengths, which, in turn, generates correlated spatial variations in saturation. These results demonstrate a new functional role for color in the perception of the 3D shape of translucent materials.

## Highlights

• Color carries information used to perceive three-dimensional shape
• Translucent surfaces internally scatter some wavelengths of light more than others
• Wavelengths weakly scattered carry information about surface orientation
• Wavelengths strongly scattered carry information about surface curvature

## Teaser

Most work on the perception of three-dimensional shapes has focused on grayscale images of achromatic surfaces. Marlow et al. show that color plays a functional role in shape perception of translucent surfaces. The physics of subsurface scattering causes saturation to vary in similar ways to the shading patterns of opaque surfaces.

## Results

**Figure 1:**
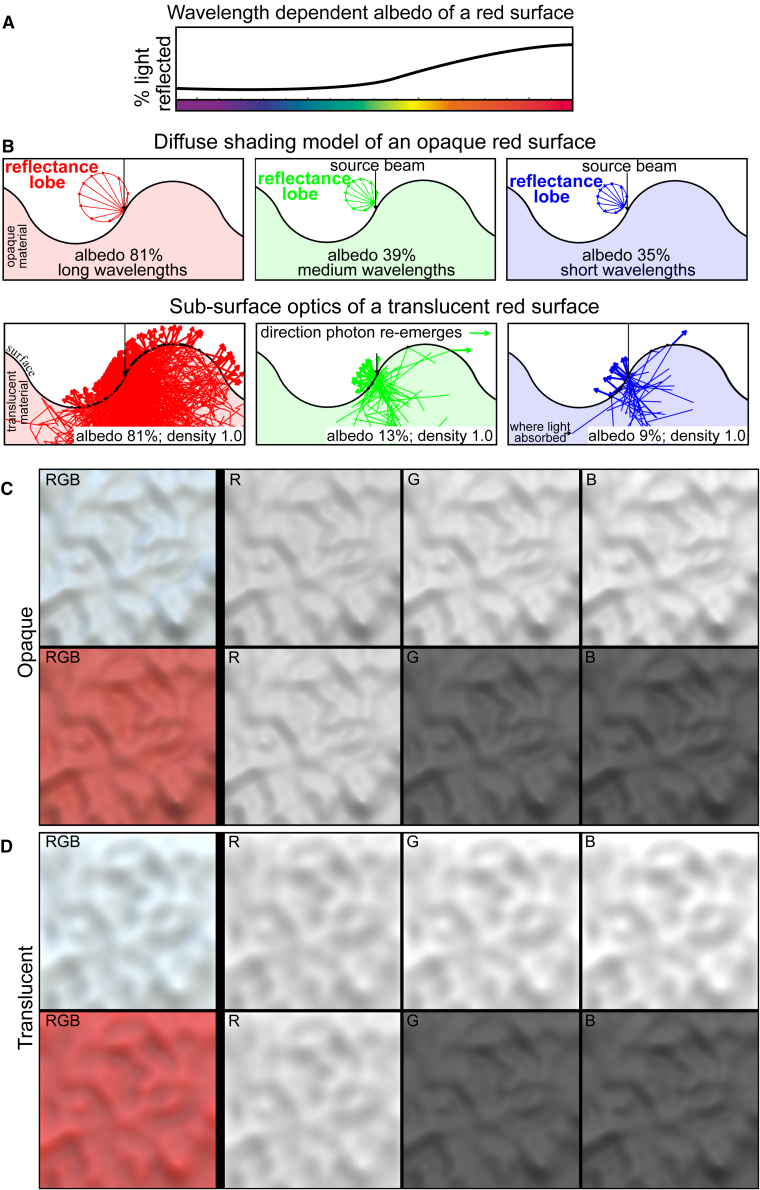
Color interacts differently with opaque and translucent materials and enhances the perceived 3D shape of translucent surfaces (A) When light interacts with a colored material, the % of light reflected in each wavelength (“albedo”) depends on wavelength. (B) The diagrams depict the diffuse reflectance lobe of opaque, shaded surfaces and the subsurface optics of translucent surfaces. A narrow beam of illumination is aimed at a point on the surface that is midway between a convex “bump” and a concave “valley.” Increasing albedo causes more light to re-emerge from both the opaque and translucent surface; however, albedo also increases the distance that light spreads beneath the translucent surface before re-emerging, often from locations remote from where it penetrated the material. (C) Colored opaque surfaces exhibit similar spatial structure in different wavelength bands, as can be seen in their RGB images; only the intensity changes. Note that the perceived 3D shape and spatial structure are similar between the chromatic and achromatic variants of the opaque surface (these effects are more pronounced if the images are viewed significantly larger than they appear at 100% resolution). (D) Colored translucent surfaces exhibit different spatial structures that depend on their albedo. The bright R channel has the highest albedo and elicits weak percepts of 3D shape. The dark channels (G and B) contain a spatial structure similar to diffuse shading, which enhances the apparent 3D shape of the red translucent surface relative to an achromatic variant rendered light gray. Note that the 3D shape of the red surface appears nearly identical to the shape of its G and B channels. The images in (C) and (D) were rendered in Maxwell Render (Next Limit Technologies), whereas the diagrams in (B) were created using a Monte Carlo simulation of subsurface scattering written in MATLAB (MathWorks). See also Figure S1.

We experience different materials as possessing different colors, which are determined by the spectral filtering properties of the pigments they contain. Achromatic pigments reflect all wavelengths equally in some fixed proportion and hence can be characterized by a single albedo (or lightness). Chromatic pigments reflect some wavelengths more than others, which means that each wavelength has an associated albedo (Figure 1A). Chromatic materials are experienced as having specific hues, lightnesses, and saturations, which depend on the proportions of different wavelengths a material emits.

Materials also differ in how permeable they are to light. Light penetrating “opaque” materials is only transported within their superficial layers, where it is scattered and re-emerges close to where it entered. For a fixed illumination, the amount of light striking a given point on a surface depends on its local orientation relative to the direction of illumination, which generates the patterns of surface shading: the amount of light returned from a point is a fixed proportion of the amount of incident light.^1^ For diffuse, uniformly colored surfaces, the proportion of light reflected in each wavelength band is fixed. Consequently, the same pattern of shading is generated by all wavelength bands, differing only by a multiplicative scale factor (Figures 1B and 1C).

Here, we show that the same is not true for translucent materials. Consider the chromatic and achromatic variants of a translucent surface depicted in Figure 1D. The only difference between these images is that the pigments within the material were rendered as either achromatic (“light gray”) or chromatic (“red”). It is immediately apparent that the perceived shape of the “red” surface is more compelling than that of its achromatic equivalent, which means that there must be more information about 3D shape in the chromatic surface than in the achromatic surface. This can be seen in the surface’s three color channels (RGB): the R channel of the “red” surface has the same structure as all three channels of the achromatic surface, but the G and B channels are different. The simulations and experiments described in what follows were designed to determine the cause of these differences.

There are four physical parameters that affect the light returned by (spatially homogeneous) translucent materials:^11^ the refraction of light when it enters or exits the surface boundary; the likelihood that light is scattered or absorbed each time it collides with pigment particles (i.e., albedo); the density of pigment particles, which determines the “mean free-path length” or average distance light travels between collisions; and the shape of the scattering distribution, which can be biased toward either forward-scattering or backward-scattering (the “phase function”). We held the refractive index, density, and phase function fixed. The refractive index was 1.3, which is characteristic of water. Our simulations revealed that the density of subsurface pigments and the phase function had negligible effects on the spatial structure in the different wavelength bands; therefore, we used an intermediate density and an isotropic phase function in both our simulations and rendering (Figures S1 and S2).

In order to assess how the reflectance properties of pigments affect the optical structure generated by subsurface scattering, we simulated the light transport processes inside of a translucent “red” bumpy surface illuminated from the front hemisphere (similar effects are observed for other colors; Figure S3C). For simplicity of exposition, we focus on RGB methods of image construction; however, it should be noted that the same arguments apply to hyperspectral images (i.e., images formed by a continuous distribution of wavelengths observed in natural scenes).

The red surface used in our experiments contains pigments that predominantly reflect long wavelengths of light, which allows them to undergo many collisions prior to exiting the surface, often at locations remote from where they entered (Figure 1B, lower left). Unlike surface shading, the intensity distributions generated by subsurface scattering are shifted away from surface normals directed at the illumination toward convexities and concavities: convex regions tend to be bright, whereas concave regions tend to be dark.^10^ Note that the R channel of the “red” surface generates the same spatial structure as all three color channels of the achromatic surface (Figure 1D).

However, consider the structure contained in the G and B channels of the red surface, which differs significantly from that of the R channel. Light at these wavelengths undergoes less subsurface transport and internal scattering, as any light that travels deeply into the material has a high probability of being absorbed. The only light that manages to escape are photons scattered backward after penetrating the superficial layers of the surface (Figure 1B, middle and right). This causes the spatial structure in these wavelength bands to behave similar to shading, which depends primarily on the intensity of incident light at a point (which is determined by local surface orientation relative to the light source; Figure 1D, lower middle and right).

Our informal observations suggest that the enhanced perception of 3D shape observed with chromatic translucent materials is due to the shading-like structure in the weakly reflected wavelength bands. This can be seen by comparing the perceived shape of the red surface with the perceived shape elicited by its G and B channels; they appear similar, if not identical. If the shading structure in these channels is indeed responsible for the enhanced perception of 3D shape in chromatic translucent materials, then the clarity of perceived 3D shape should scale with the shading contrast generated in these wavelength bands. It is known that the contrast of diffuse shading is weakest when surfaces are illuminated along the axis of surface relief, and that it monotonically increases as the illumination direction becomes more oblique.^12^ This suggests that the perceived clarity of 3D shape in our chromatic stimuli should exhibit similar dependencies on illumination direction.

**Figure 2:**
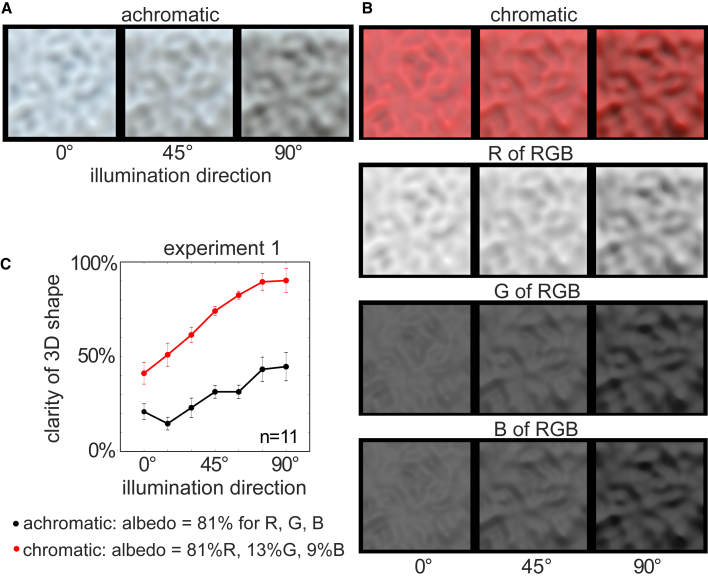
The clarity of the perceived 3D shape depends on the contrast of color channels with low albedos Experiment 1 parametrically varied the primary illumination direction from along the line of sight (left column of each panel) to above the surface (right column of each panel) or between these two extremes (midpoint is shown in the middle column of each panel). (A) A light-gray translucent surface characterized by deep patterns of subsurface scattering. (B) A red translucent surface. The second row depicts the R RGB channel of the red surface, and the bottom two rows depict the G and B channels of the red surface, respectively. The benefit of color for shape perception generalizes across different directions of illumination and is strongest for illumination directions that generate high-contrast shading structure in the G and B channels. (C) The graph plots the proportion of trials that observers selected each surface as having a clearer 3D shape than the other 13 surfaces in experiment 1. Error bars are standard errors of the mean of 11 observers. See also Figures S2 and S3.

Experiment 1 was conducted to test this prediction by manipulating the primary illumination direction of a natural light map (i.e., a map of light sources in a real-world scene) of achromatic (“light gray”) and chromatic (“red”) surfaces. Observers viewed the surface along a direction perpendicular to the plane. The light map was rotated relative to the dominant illumination direction to create 7 different illumination conditions, which ranged from along the line of sight to directly above the surface (i.e., 90° above the viewing direction; STAR Methods; Figures 2 and S3A). Note that the change in the illumination direction only has a small effect on the structure generated by achromatic translucent surfaces; the main change is an increase in contrast of the convexities and concavities. However, the same is not true for the shading-like structure in the G and B color channels of the red surface. The different illumination directions generate different spatial patterns of shading, and at low elevations, its contrast—and the perception of 3D shape in the G and B channels—is nearly eliminated.

The results shown in Figure 2C support the hypothesis that the perceived 3D shape depends on the shading-like structure in the G and B channels. Observers viewed every pair of the 14 surfaces and judged which ones elicited a clearer percept of 3D shape. Although the chromatic surface always has a clearer 3D shape than the achromatic surface (F_(1,10)_ = 186; p < 0.01), perceived 3D shape becomes clearer as the illumination direction approaches light-from-above (F_(1,10)_ = 13.4; p < 0.05), which is also associated with the largest effect of color on perceived shape (F_(1,10)_ = 7.5; p < 0.05). Note that the clarity of the shape of the achromatic surface only approaches the level of the weakest perception of 3D shape in the chromatic surface when the achromatic surface has maximal contrast (illumination-from-above), and the shading structure is largely eliminated in the B and G channels (i.e., when illuminated along the axis of surface relief).

**Figure 3:**
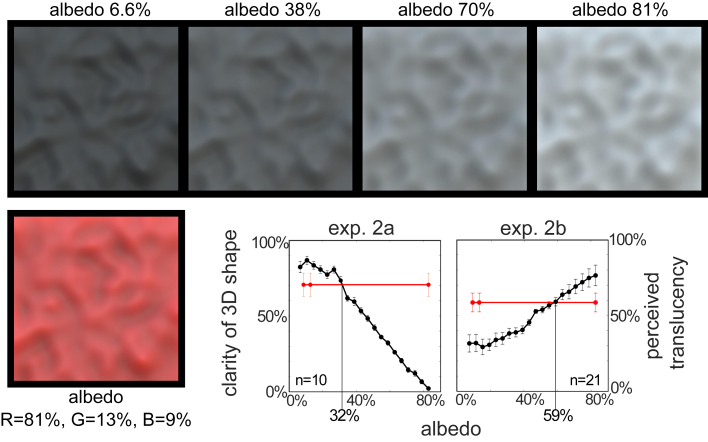
The albedo of achromatic translucent surfaces affects the spatial pattern of returned light and perceived 3D shape The top row depicts achromatic surfaces rendered with different albedos. The optical structure generated by darker surfaces is more similar to shading than that of lighter surfaces (for a fixed density of pigment particles suspended in the material) because light penetrates the surface less deeply. Left graph (experiment 2a): the proportion of trials that each surface was selected as having a clearer 3D shape is plotted against the reflectance of the subsurface pigments (the red surface has three values along the x axis because it has different albedos in the different wavelength bands used to generate RGB images). Right graph (experiment 2b): the results using the same images where observers judged which surface appeared more translucent. Note that the points of subjective equality (PSE) of the two judgments are very different, which suggests that it is not possible to match both the 3D shape and material properties of the red surface with a single (achromatic) albedo. Error bars are SEM. See also Figure S3.

The multiple albedos of our colored surfaces generate different forms of spatial structure that may play different roles in the perception of translucency and 3D shape. If so, it should not be possible to equate both shape and material of chromatic and achromatic surfaces. Our informal observations suggest that most conventional conversions of chromatic translucent materials to grayscale^13^ result in weaker percepts of both 3D shape and perceived translucency (Figure S3D), suggesting that such a transformation results in the loss of information about both shape and material. However, it is unclear whether this is simply due to the particular conversions we tried. In experiment 2, we addressed this question by creating 20 different albedos of our achromatic surface while fixing all other variables to those of the red surface (Figure 3). The primary illumination direction was 45° above the surface and the observer’s viewing direction. Observers judged the clarity of the perceived 3D shape in experiment 2a and the perceived translucency in experiment 2b. Observers viewed every pairwise combination of 20 different levels of achromatic albedo and the red surface used in experiment 1. We reasoned that if the percepts of 3D shape and material evoked by our red stimulus can be matched with an appropriately chosen achromatic stimulus, then the points of subject equality (PSEs) of both 3D shape and translucency judgments should occur at the same level of achromatic surface albedo.

The results indicate that there is no achromatic surface albedo that appears equally 3D and translucent as the chromatic surface. The proportion of trials where each surface was selected as having a clearer 3D shape is plotted against the albedo of the subsurface pigments (Figure 3, left graph). The results demonstrate that the dark albedos, which generate shallower amounts of subsurface scattering and more shading-like image structure, evoke a clearer percept of 3D shape than light albedos (F_(1,9)_ = 1,949; p < 0.01). The PSE of the shape judgments for chromatic and achromatic stimuli occurs at an albedo of ∼32%. The results of experiment 2b exhibit the opposite trend (F_(1,20)_ = 16.4; p < 0.01): perceived translucency increases as a function of pigment albedo in the achromatic surface, with a PSE of 59%. Taken together, the results of experiments 2a and 2b suggest that it is not possible to equate both the clarity of the perceived 3D shape and the perceived translucency of chromatic and achromatic surfaces. Similar results were observed in a control experiment that multiplicatively scaled the image intensity to equate the brightest points in all stimuli (Figure S3).

## Discussion

The simulations described earlier demonstrate that the wavelength-specific reflectance of pigments suspended in translucent materials causes different degrees of subsurface transport and scattering, which, in turn, generates different forms of spatial structure in RGB color channels. Our experiments suggest that (1) shading structure is primarily generated at wavelengths with low albedos, and (2) color provides information about shape and/or material that cannot be “matched” by any single albedo of an achromatic surface. For ease of exposition, we focused on RGB methods of image construction to demonstrate clear links between the spatial structure in discrete color channels and reflectance in those wavelength bands. However, it is clear that the visual system does not have any direct access to the content of RGB color channels;^14^, ^15^, ^16^, ^17^, ^18^ information on these channels must somehow be expressed in different dimensions of color that humans experience: hue, saturation, and brightness (“value” in HSV color space and “lightness” in CIELAB space).^18^, ^19^, ^20^

**Figure 4:**
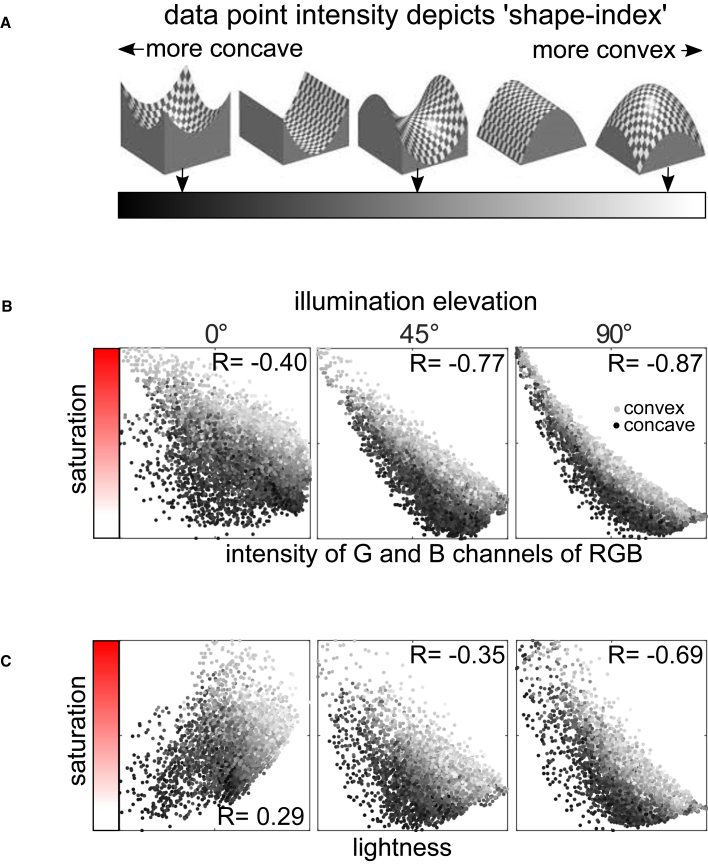
The pattern of saturation across our surfaces is linked to two forms of 3D shape information that could theoretically support shape and material perception The RGB image of the red surfaces in Figure 2B was transformed into CIELAB (1972) color coordinates. Lightness is a nonlinear monotonic transformation of luminance, and saturation is the radius of the color coordinate from the origin of the a,b plane, relative to its lightness. (A) Different shades of gray distinguish pixel values originating from concave, saddle, and convex surface regions (i.e., “shape index”^21^). (B) Saturation covaries with the intensity of the B and G channels of the red surface, which exhibit a shading-like structure. Note that as illumination elevation increases, the strength of the covariance also increases and is in line with our psychophysical data (see Figure 2C). Note also that the residuals in these plots are systematic: saturation is also linked to the local sign of surface curvature, particularly at low illumination elevations. (C) The relationship between lightness and saturation for our surfaces can vary dramatically depending on the illumination direction. The R values show Spearman correlation coefficients. The correlations between saturation and shape index values are R = 0.68, 0.45, and 0.26 for the three illumination elevations. The proportion of variance R^2^ in saturation accounted for by a multilinear regression of G and B and shape index with the three illumination directions is: 0.73, 0.85, and 0.90. Less variance in saturation is accounted for by lightness and shape index: 0.46, 0.66, and 0.76. See also Figure S3 and Table S1.

There are two forms of spatial structure generated by our chromatic (“red”) stimulus: variations that behave similar to surface shading (carried predominantly in the B and G channels) and variations that are constrained by the direction of curvature (the R channel). Our experiments suggest that when shading is present, it dominates our perception of 3D shape. The goal of the analyses presented in Figure 4 was to assess whether the shading structure in these channels manifests as variations in saturation, brightness, or both (the hue variations in our chromatic stimulus were negligible).

We addressed this question by plotting the saturation and brightness of the pixels in our “red” stimulus as a function of the intensity in its G and B channels. In these plots, the contribution of curvature was visualized by coding the intensity of each surface pixel based on its local “shape index^21^” (Figure 4A; STAR Methods). Smooth surfaces contain only three qualitative shapes: convexities, concavities, and saddles. In our analysis, surface pixels that are symmetric concavities are coded as black data points, symmetric convexities are coded light gray, and symmetric saddles are midway between these two values. The other values vary as a function of the symmetry of their principal axes of curvature (Figure 4A; STAR Methods).

Our analysis reveals that shading structure correlates with saturation: darker pixels in the G and B channels are more saturated than brighter pixels. This makes intuitive sense; regions that are “dark” in B and G generate relatively saturated reds, whereas regions that are relatively “bright” in B and G will generate less saturated reds (this is the definition of saturation or “purity” of a hue). The strength of this covariation increases monotonically with the shading contrast in these channels, consistent with our psychophysical results in experiment 1.

The plots in Figure 4B also reveal that the direction of curvature (depicted by the intensity of each data point) also generates systematic variance: the saturation of concave regions is generically lower than convexities and decreases more rapidly as the intensity in the G and B channels increases (i.e., has steeper slopes). Thus, these analyses reveal that the spatial variations in saturation generated by subsurface scattering contain information about both surface orientation (shading) and the direction of surface curvature.

Previous work on transparent surfaces has suggested that colored materials illuminated from behind will appear more saturated and darker than thinner regions of the same material, which has also been observed for translucent materials.^22^^,^^23^ In our experiments, we prevented light that enters from the rear to be emitted from the front by using thick surfaces and restricting the primary direction of illumination to the front hemifield. In such conditions, the covariation of saturation and brightness is relatively weak and unstable; correlations were weakly positive for frontal illumination and became negative as the illumination direction became more oblique (Figure 4C). Our results show that saturation covaries with the intensity of the G and B channels (Figure 4B) more than with overall image brightness (Figure 4C), which implies that it is predominantly providing information about surface shading, not surface thickness. Thus, our work provides new insight into the information that can be conveyed by spatial variations in saturation and brightness.

What role does the direction of curvature play in the perception of translucent stimuli? Experiment 2 showed that it is not possible to match both the perceived 3D shape and opacity of achromatic and chromatic surfaces. The results suggest that shading information, when present, dominates the perception of 3D shape. Indeed, the perceived 3D shape of our “red” surface is essentially identical to that experienced when viewing its G and B channels. However, achromatic surfaces that generate equivalent percepts of 3D shape appear substantially more opaque than their chromatic match. This suggests that the spatial variations in saturation linked to the direction of surface curvature provide information about the material properties of translucent surfaces.

Previous work has shown that the visual system exploits the covariation of achromatic intensity and color to distinguish changes in reflectance from illumination.^7^, ^8^, ^9^ Changes in surface reflectance typically cause simultaneous changes in hue and intensity, whereas changes in the amount of illumination are largely independent of hue. Studies have shown^7^, ^8^, ^9^ that when changes in hue are aligned with changes in intensity, variations in intensity are harder to detect and the perception of shape from shading is weak; however, when variations in intensity are misaligned with changes in hue, intensity gradients are more likely to appear as shading, enhancing the perception of 3D shape. In these studies, chromatic variations did not provide any direct information about 3D shape; they modulated the likelihood that changes in intensity were interpreted as shading.

The studies reported here demonstrate that spatial variations in color—or more specifically, chromatic saturation—can directly enhance the perception of 3D shape. We further showed that it is not possible to match both the perceived 3D shape and the perceived opacity of chromatic translucent materials with achromatic materials containing only one albedo. Taken together, these results provide new insights into the role of color in the perception of 3D shape and the material properties of translucent surfaces.

## STAR★Methods

### Key resources table

**Table:** 

REAGENT or RESOURCE	SOURCE	IDENTIFIER
**Deposited data**		

Psychophysical Data	This paper	https://doi.org/10.17632/w2k76w467w.1

**Software and algorithms**		

SSSfig1.m	This paper	https://doi.org/10.17632/w2k76w467w.1

**Other**		

Maxwell Scene Files ^∗^.mxs	This paper; Mendeley Data	https://doi.org/10.17632/w2k76w467w.1
3D surface geometry: Triangulated Mesh ^∗^.obj	This paper; Mendeley Data	https://doi.org/10.17632/w2k76w467w.1
Light map: ^∗^.hdr	This paper; Mendeley Data	https://doi.org/10.17632/w2k76w467w.1

### Resource availability

#### Lead contact

Further information and requests for resources should be directed to and will be fulfilled by the lead contact, Phillip Marlow (phillip.marlow@sydney.edu.au).

#### Materials availability

Scenes used to render images generated for this study have been deposited to Mendeley. Mendeley Data: https://doi.org/10.17632/w2k76w467w.1.

### Experiment model and subject details

All observers that participated in the experiments were undergraduate psychology students enrolled in either first or second year psychology courses at the University of Sydney. They received a small amount of course credit for their participation. They had no knowledge of the hypotheses. Informed consent was obtained from all observers and the experiment was approved by the Human Research Ethics Committee at the University of Sydney in accordance with the Declaration of Helsinki.

### Method details

#### General methods

All images in this paper depicted the same 3D shape: a smooth bumpy plane oriented fronto-parallel to the observer. A triangulated mesh (OBJ format) describing the bumpy plane’s 3D surface geometry is available at Mendeley [Mendeley Data: https://doi.org/10.17632/w2k76w467w.1].

The material properties of the surface were ray traced using software that is designed to have high physical accuracy for translucent substances - Maxwell Render (Version 3; Next Limit Technologies). Six parameters control the material properties of translucent media in Maxwell Render: surface roughness; refractive index; attenuation; phase function; particle reflectance; and particle density. Surface roughness, refractive index and phase function were held fixed. Surface roughness was set to 100, which eliminates specular reflection; refractive index was set to 1.3, which was chosen because many natural translucent materials have a high water content; phase function was to 0, which means the direction of sub-surface scattering was isotropic (no bias for forward-scattering versus back-scattering). The values of the other three material parameters varied from experiment to experiment are given below in the method section of each experiment.

The images were rendered using image-based lighting (IBL). The IBL was a panoramic photograph (a ‘light map’) of a grove of Eucalyptus trees at UC Berkeley (downloaded from http://gl.ict.usc.edu/Data/HighResProbes/). The images were rendered using orthographic perspective.

DOI accession numbers of the scene files (.mxs) are given in the key resources table and fully describe the camera, 3D shape, material, and illumination. The colorspace of the rendered images is Standard RGB IEC 61966-2-1:1999. Observers viewed the images online due to the cessation of face-to-face testing during COVID19 pandemic. Their personal computers were assumed to use the same Standard RGB color space; we have found that all of the effects described in the paper are robust to variations in viewing displays.

The same procedures were used in all experiments. On each trial, observers viewed two images presented side-by-side. Their task was to select the surface that appeared to have the clearest 3D surface shape. The design of the experiments was a ‘paired comparison task’ in which every image in the experiment is presented once beside every other image in the experiment. The dependent variable in each experiment is the proportion of trials that each image was selected as having a clearer 3D surface shape (in proportion to the number of trials that each image was presented/ could have been chosen as having clearest 3D surface shape).

#### Experiment 1

11 observers viewed fourteen images of the smooth bumpy plane. Seven of the images were rendered with a light grey translucent material and the other seven were a red material. The material parameters of the light grey and red surfaces were set to: attenuation (4.29mm); particle density (305.41); and particle reflectance differed between the white and red surfaces. The light-interacting particles suspended in the light grey variant scattered 81% of light-rays and absorbed 19%. The red variant of the same surface was constructed by assigning different values of light-scatter and reciprocally light-absorption as a function of wavelength. Specifically, wavelengths associated with blue scattered 9% and hence absorbed 91%; green scattered 13% (absorbed 87%); and red scattered and absorbed light identically to the light grey surface (scattered 81%). Hence, the red color channel of RGB images is nearly identical to that of all three color channels of the RGB image of the white surface. The white and red surfaces were illuminated from seven different equally spaced directions spanning a range from frontal illumination at one extreme to light-from-above at the other extreme. Illumination direction was varied by rotating the light map that illuminated the surfaces.

The design of the experiments was a ‘paired comparison task’ in which every image in the experiment is presented once beside every other image in the experiment. On each trial, observers viewed two images presented side-by-side. Their task was to select the surface that appeared to have the clearest 3D surface shape. The results shown in Figure 2C depict the proportion of trials that each image was selected as having a clearer 3D surface shape (in proportion to the number of trials that each image was presented/ could have been chosen as having clearest 3D surface shape).

#### Experiment 2

Observers viewed 21 images of the smooth bumpy plane (n=10 in Exp. 2a and n=21 in Exp. 2b). 20 were rendered with achromatic material and the albedo of the subsurface pigments was varied. One was the same red material used in Experiment 1. The density (305.41) of the pigment particles was held fixed and hence the albedo of the particles was the only parameter that varied. The twenty values of albedo chosen using Maxwell Render’s interface (in which albedo varies from 0 to 255) were 17, 27, 37…207. The primary illumination direction was held fixed at a 45° elevation bisecting the viewing direction and vertical axis. The design of the experiments was also a ‘paired comparison task’ in which every image in the experiment is presented once beside every other image in the experiment. Observers viewed two surfaces on each trial and judged which had either clearer 3D shape (left graph of Figure 3) or appeared more translucent (right graph of Figure 3). Different groups of observers were used for Exp. 2a and 2b.

#### Sub-surface simulation

The paths of the light-rays depicted in Figure 1 were calculated using a Monte Carlo ray tracer written in MATLAB (R2021a) by the first author. The surface geometry was composed of cylindrical segments forming an alternating pattern of convex ridges and concave valleys (radius = 1.1148; centre-to-centre separation of cylinders = 2.0268). The surface was illuminated by a beam of light; all 200 photons entered the surface at a single location and along the same vertical direction. The entry point was the inflection point between the convexity and concavity. Because geometry was perfectly circular, the intersection of light rays with the surface was solved using line-circle intersection, which is less computationally time consuming than line-triangle intersection. Refraction, internal reflection and Fresnel effects (Schlick’s approximation) were implemented using formulas described by Degreve [http://www.flipcode.com/archives/reflection_transmission.pdf]. The refraction indices of the material and its optical medium were 1.3 and 1.0 (respectively), which were chosen to approximate water and air. The surface was perfectly smooth. Because our goal was to illustrate the effect of albedo on the extent of sub-surface light transport, the simulation ignored the component of light that would be specularly reflected by such a material. Only light refracting into the material was traced. The first sub-surface event occurred at a randomly selected distance along the refracted beam. Specifically, the free-path-length of photons (the distance between sub-surface events) was drawn from an exponential distribution, P=0.001−αlogβ. Where P is path length, 0≥β≤1 drawn from a uniform random distribution, and α is the inverse of ‘density’ and was set to 0.5. Each sub-surface event involved deciding whether the photon was absorbed, which depends on the albedo of the participating medium. If the photon was not absorbed, a random 3D direction of internal scatter was chosen and a new free-path-length *(P)* was chosen to move along this new direction. If that path intercepts the surface, then the photon may re-emerge or be internally reflected back into the material. The simulation calculated the probability of re-emergence versus internal reflection, which is a function of the angle of incidence and refractive indices.

### Quantification and statistical analysis

The results were analyzed using a multivariate analysis of variance with planned orthogonal contrasts. The Decision-Wise error rate (*α* = 0.05) was controlled for each of the contrasts. In Experiment 1, we tested for a main effect of chromatic versus achromatic, linear trend as a function of elevation, and their interaction. In Experiment 2, we tested for linear trend as a function of albedo.

In Figure 4, qualitatively different local 3D shapes were distinguished using a ‘shape index’^21^ statistic that varies from -1 to 1. Shape index is a statistic that is designed to capture different types of curvature and in a way that is independent of the amount of curvature. Hence, a sphere has the ‘shape index’ value (-1) independently of the size of the sphere (i.e., its curvature radii/ amount of curvature). Shape index is calculated using the maximum and minimum surface curvature - k_1_ and k_2_ – arranged such that k_1_> k_2_. Specifically, shape index is equal to

$$\mathrm{atan}2\left(\frac{\left({\text{k}}_{2}+{\text{k}}_{1}\right)}{\left({\text{k}}_{2}-{\text{k}}_{1}\right)}\right)$$

Surfaces that have negative shape index values are convex in the direction that they curve most rapidly, whereas surfaces that have positive shape index values are concave in the direction of most rapid curvature. Shape index values near zero are saddle shapes that curve convex in one direction and concave in the other direction.

The analyses presented in Figure 4 and Table S1 were performed on downsampled versions of our images (67x67 pixels) to reduce the number of data points visible in the graphs; however, we found that the results were extremely similar for higher image resolutions.

Saturation, and ‘lightness’ were calculated in CIElab color space. (Note that ‘lightness’ in CIElab is a non-linear monotonic transformation of luminance; the same R value for the rank-order correlations would be obtained for both luminance and ‘lightness’). Our images were converted into to this color space using the matlab function rgb2lab with the input option set to ‘sRGB’ to match the format used by our ray-tracing software (Maxwell Render v3). In CIElab, saturation is equal to:

$$\frac{\sqrt{{\text{a}}^{*2}+b^{*2}}}{L^{*}}$$

where $L^{*}$ (‘lightness’), ${\text{a}}^{*}$, and $b^{*}$ are CIElab coordinates of a surface pixel.

The correlations reported in our paper are Spearman ‘rank-order’ correlations chosen because they better capture the nonlinear associations in our data.

Table S1 depicts separate correlations between saturation and the intensity of either the G or B channel. The range of the values in each channel was normalized to a range of 0 to 1. The correlations in Figure 4 between saturation and the G and B channel combined the intensities of the G and B. This was done by concatenating the array of G and B intensities (after normalizing the range of each).

## Data Availability

• All data have been deposited at Mendeley and are publicly available as of the date of publication. Mendeley Data: https://doi.org/10.17632/w2k76w467w.1. • Any additional information required to reanalyze the data reported in this paper is available from the lead contact upon request. • Original code written to simulate sub-surface scattering have been deposited to Mendeley Data: https://doi.org/10.17632/w2k76w467w.1.

## Supplemental information

Document S1. Figures S1–S3 and Table S1

Document S2. Article plus supplemental information
