# Supplementary material for: The role of color in the perception of three-dimensional shape
Source: Curr Biol. 2022 Mar 28;32(6):1387–1394.e3. doi: 10.1016/j.cub.2022.01.026 (PMC8967406; doi:10.1016/j.cub.2022.01.026)
Supplement: Document S1. Figures S1–S3 and Table S1 [file mmc1.pdf]

Current Biology, Volume *32*

Supplemental Information

The role of color in the perception of three-dimensional shape

Phillip J. Marlow, Karl R. Gegenfurtner, and Barton L. Anderson

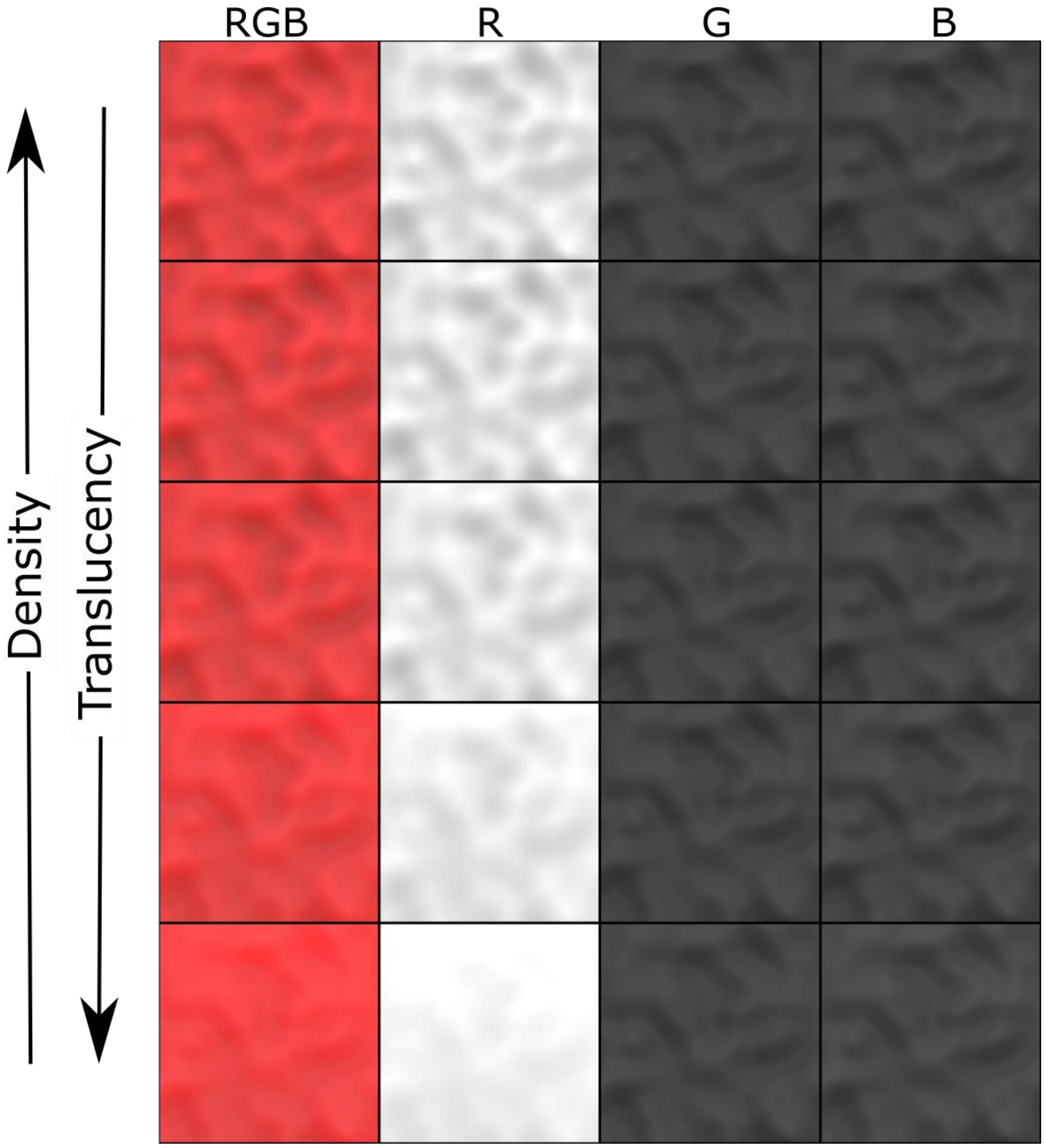

Figure S1. The effect of color on shape generalizes to translucent materials rendered with different densities of sub-surface pigments. Related to Figure 1D. The figure depicts the same 3D shape rendered with a primary illumination direction 45˚ above the observer. The only parameter that varies is the density of the pigments suspended in the material, which causes the surface to appear more translucent for lower densities. Density mainly affects the contrast of the R channel. Shading-like structure exists in the G and B channels of the red surface for all densities tested. Note that perceived 3D shape is clearer for the red surface than for the R channel for all densities. We performed the same analysis described in the general discussion to assess whether 3D shape information exists in the pattern of saturation across the surfaces. A multilinear regression showed that most of the variance in saturation can be accounted for by a linear combination of local sign of curvature and the intensity of the B and G channels. The R^2 values of the multilinear regression for the 5 surfaces were (given in descending order of density): 0.92, 0.90, 0.88, 0.86, 0.88. The shading-like structure in the G and B channels dominates the spatial pattern of saturation over these surfaces. The correlation coefficient (Spearman R value) for saturation correlated with either the intensity of the B and G channels or local sign of surface curvature (given in brackets) was: -0.88(0.07), -0.81(0.17), -0.83(0.09), -0.87(-0.06), -0.92(-0.15).

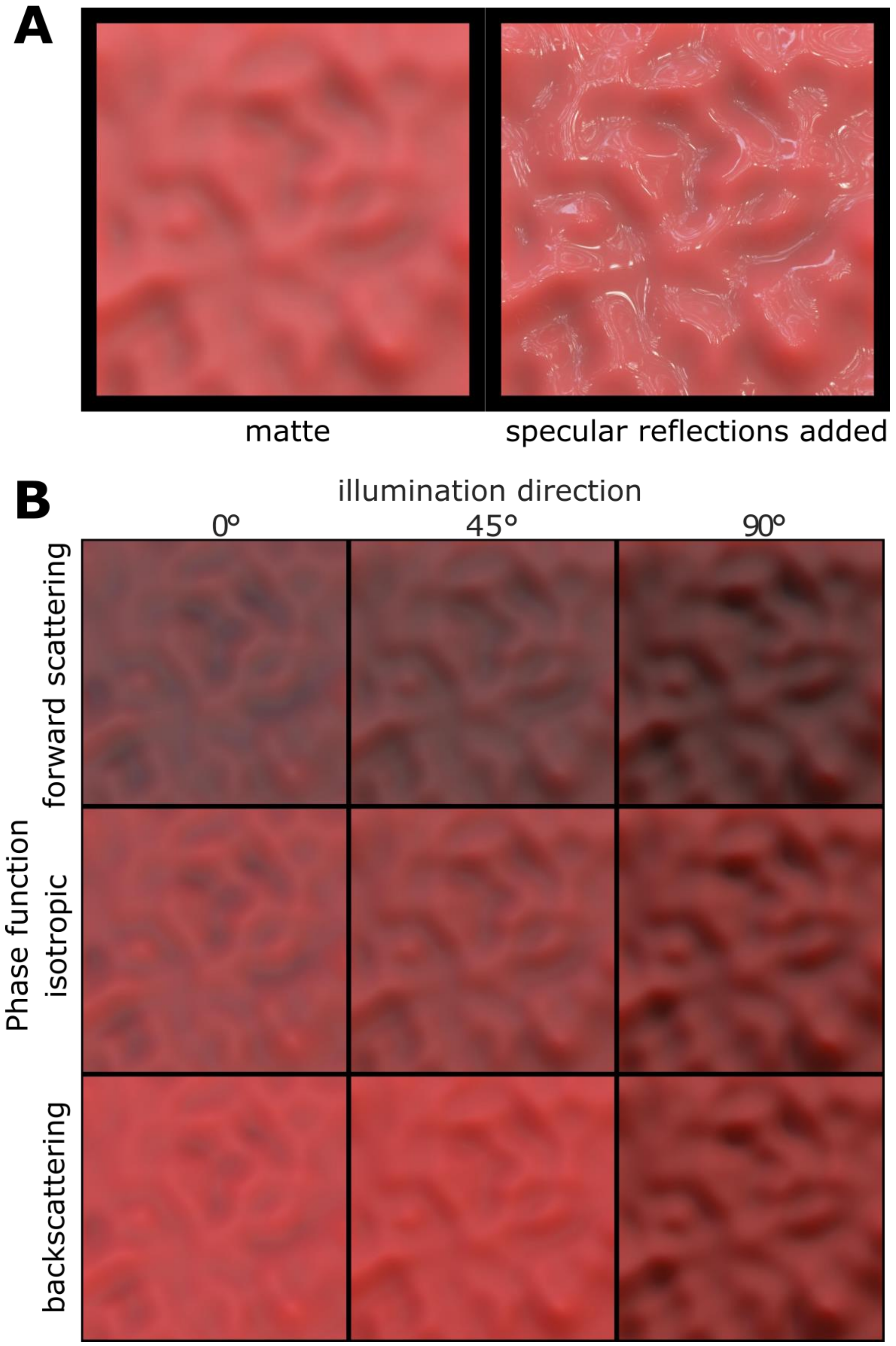

Figure S2. Effects of specular reflections and biased sub-surface scattering distributions on realism and clarity of 3D surfaces. Related to Figure 2. A: Adding specular reflections increases the apparent realism, translucency, and vividness of perceived 3D shape. The surfaces in our experiments were rendered without specular reflections in order to study effects of the color of sub-surface scattering on apparent 3D shape. **B:** The surfaces used in our study were rendered with isotropic sub-surface scattering. The top and bottom row are variants rendered with either forward scattering or back scattering. Forward scattering sends light deeper into the material. Hence, light is less likely to re-emerge and the surface appears darker. Likewise, backscattering has the opposite effect; more light re-emerges and the surface appears brighter. The effects of the scattering

distribution on perceived 3D shape are slight compared to the large differences in brightness and effect of illumination direction. We performed the same analysis described in the general discussion to assess whether 3D shape information exists in the pattern of saturation across the surfaces. A multilinear regression showed that most of the variance in saturation can be accounted for by a linear combination of local sign of curvature and the intensity of the B and G channels. The R^2 values of the multilinear regression for the 9 surfaces in **B** were (given in row-major order): 0.65, 0.78, 0.85, 0.71, 0.86, 0.91, 0.81, 0.95, 0.95. We observed that the scattering distribution mainly affects the bright 'R' channel, whereas the illumination direction mainly affects the contrast of the 'G' and 'B' channels that carry shading-like structure. Specifically, the 'R' channel has higher contrast when forward scattering phase functions send light deeper into the material, whereas the G and B channels have higher contrast at higher elevations of the primary illumination direction. These variations in contrast affect whether saturation depends more on local sign of surface curvature or local 3D surface orientation. Saturation correlates more strongly with local sign of curvature when either forward scattering increases the contrast of the R channel or when frontal illumination reduces the contrast of the shading-like structure in the G and B channels. The opposite occurs when either illumination or material properties increase the contrast of shading-like structure or decrease the contrast of the R channel. Saturation correlates more strongly with the intensity of the B and G channels when those channels have higher contrast in the light-from-above illumination condition. Likewise, saturation correlates more strongly with the intensity of the G and B channels than local sign of curvature when backscattering reduces the contrast of the 'R' channel. The correlation coefficient (Spearman R value) for saturation correlated with either the intensity of the B and G channels or local sign of surface curvature (given in brackets) were: -0.18(0.74), -0.54(0.52), -0.79(0.31), -0.38(0.67), -0.69(0.40), -0.88(0.21), -0.65(0.46), -0.92(0.09), -0.96(0.07).

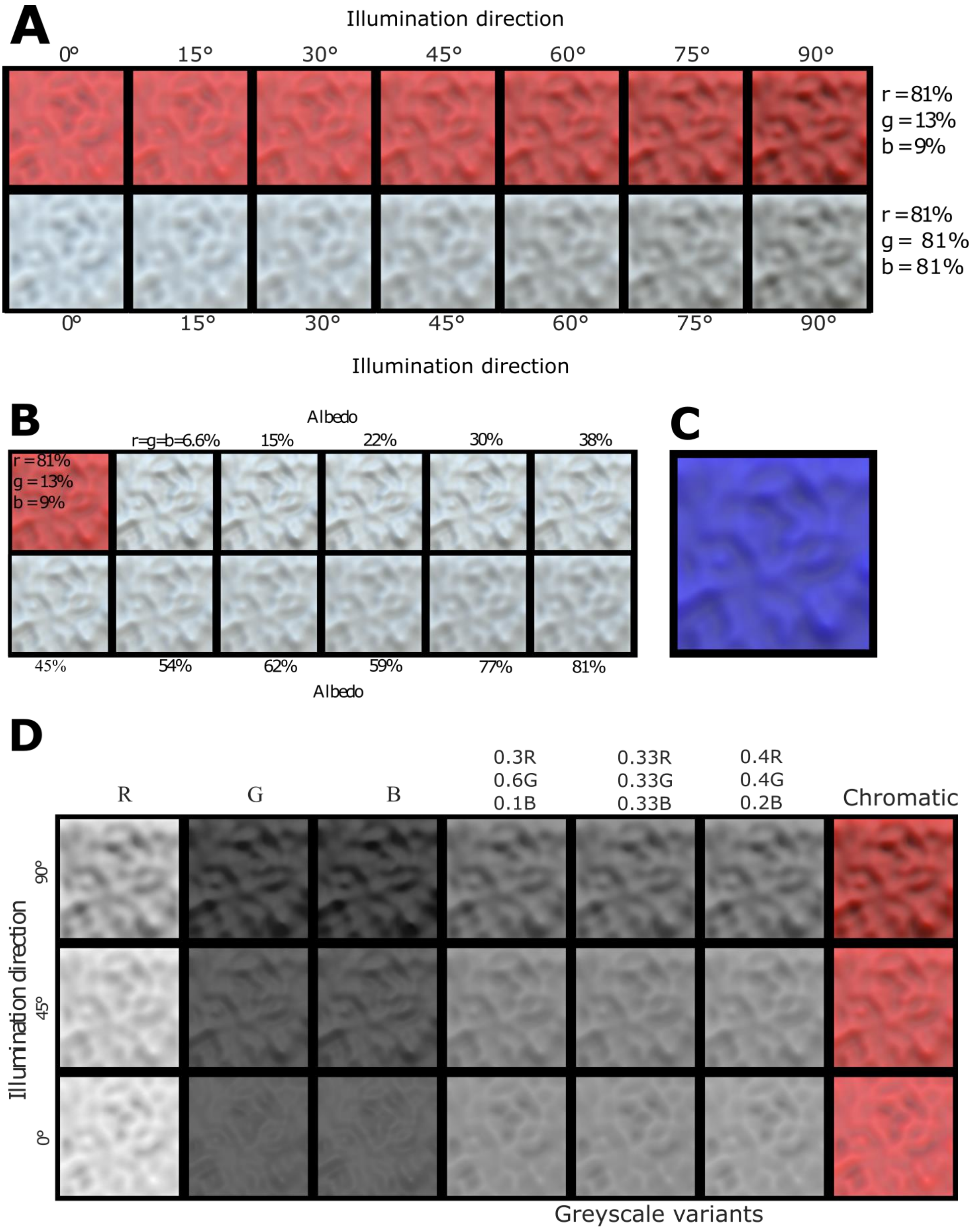

Figure S3. The effect of illumination direction, albedo, color, and grayscale conversion on surface appearance. Related to Figure 2 and 3. A: The 7 levels of illumination direction used in Experiment 1. **B:** The same surfaces as in Figure 3 except the max. intensity of the images has been equated (by scaling intensity multiplicatively). The surfaces with darker albedos still have more vivid 3D shape when perceived lightness has been equated. **C:** A blue variant of the red translucent surface in Figure 1 demonstrating that our effects of color generalize to other hues. **D:** Our red surfaces exhibit more vivid 3D shape and appear more translucent than greyscale variants generated using conventional decolorisation algorithms. The left three columns depict the intensity and structure of the three RGB color channels of the red surfaces in the right-most column. Three

greyscale variants of each red surface were generated by performing a weighted average of the three color channels using the weights depicted above each column. The greyscale variants shown in the fourth column were generated using the same weights as MATLAB's rgb2gray algorithm. The fifth column was generated by assigning equal weight to R,G, and B. The sixth column was generated by assigning B a lower weight than R and G, which had equal weight. Our informal observations suggest that the only way to generate a greyscale variant that has equally clear 3D shape as the red surface is to assign large weights to the RGB channels that contain shading like structure, which dramatically reduces perceived translucency. It is unclear whether it is possible to equate both perceived 3D shape and perceived translucency between RGB and greyscale variants.

	Lightness	***Saturation***	***R of RGB***	***G of RGB***	***B of RGB***	***ShapeIndex***	***Shading***
Lightness	*1.00 (0˚)*<br>*1.00 (45˚)*<br>*1.00 (90˚)*	*0.29*<br>*-0.35*<br>*-0.69*	*0.92*<br>*0.91*<br>*0.97*	*0.76*<br>*0.88*<br>*0.95*	*0.62*<br>*0.46*<br>*0.94*	*0.70*<br>*0.46*<br>*0.33*	*0.48*<br>*0.74*<br>*0.85*
Saturation	*0.29*<br>*-0.35*<br>*-0.69*	*1.00*<br>*1.00*<br>*1.00*	*0.60*<br>*-0.06*<br>*-0.50*	*-0.34*<br>*-0.67*<br>*-0.86*	*-0.46*<br>*-0.70*<br>*-0.88*	*0.68*<br>*0.45*<br>*0.26*	*-0.60*<br>*-0.77*<br>*-0.90*
R of RGB	*0.93*<br>*0.95*<br>*0.97*	*0.60*<br>*-0.06*<br>*-0.50*	*1.00*<br>*1.00*<br>*1.00*	*0.48*<br>*0.74*<br>*0.85*	*0.34*<br>*0.70*<br>*0.82*	*0.84*<br>*0.65*<br>*0.49*	*0.18*<br>*0.53*<br>*0.72*
G of RGB	*0.76*<br>*0.91*<br>*0.95*	*-0.34*<br>*-0.67*<br>*-0.86*	*0.48*<br>*0.74*<br>*0.85*	*1.00*<br>*1.00*<br>*1.00*	*0.93*<br>*0.98*<br>*1.00*	*0.21*<br>*0.16*<br>*0.11*	*0.87*<br>*0.89*<br>*0.94*
B of RGB	*0.62*<br>*0.88*<br>*0.94*	*-0.46*<br>*-0.70*<br>*-0.88*	*0.34*<br>*0.70*<br>*0.82*	*0.93*<br>*0.98*<br>*1.00*	*1.00*<br>*1.00*<br>*1.00*	*0.14*<br>*0.13*<br>*0.08*	*0.87*<br>*0.93*<br>*0.95*
ShapeIndex	*0.70*<br>*0.46*<br>*0.33*	*0.68*<br>*0.45*<br>*0.26*	*0.84*<br>*0.65*<br>*0.49*	*0.21*<br>*0.16*<br>*0.11*	*0.14*<br>*0.13*<br>*0.08*	*1.00*<br>*1.00*<br>*1.00*	*0.00*<br>*-0.01*<br>*0.00*
Shading	*0.48*<br>*0.74*<br>*0.85*	*-0.60*<br>*-0.77*<br>*-0.90*	*0.18*<br>*0.53*<br>*0.72*	*0.87*<br>*0.89*<br>*0.94*	*0.87*<br>*0.93*<br>*0.95*	*0.00*<br>*-0.01*<br>*0.00*	*1.00*<br>*1.00*<br>*1.00*

Table S1. Spearman correlation coefficients summarising covariation between 3D orientation, convexity, and pixel values in RGB and CIElab coordinates. Related to Figure 4. The three rows for each pair of variables shows correlation coefficient for three illumination directions: (0˚, i.e., frontal illumination; 45 ˚; and 90˚, i.e., light-from-above). 'Shape Index' distinguishes convexities, concavities, and saddles and can be visualised in Figure 4b. The last row and column dubbed 'Shading' is an idealized form of shading representing the intensity of a Lambertian surface rendered with a single collimated light source. Specifically, it is the dot product of the surface normal and the primary illumination direction of the light map that illuminated each translucent surface. Note that saturation is strongly correlated with the shading-like structure in the G and B channels, particularly when the surfaces are illuminated from above and had clearest 3D shape. Indeed, saturation correlates slightly more strongly with the idealized form of shading than the shading-like structure in the G and B channels. This is because all three channels exhibit some dependence of shape index. Saturation only encodes differences between R,G, and B and hence factors out these shared dependencies on curvature.
